# The Clinical Applications of a Systems Approach

**DOI:** 10.1371/journal.pmed.0030209

**Published:** 2006-05-23

**Authors:** Andrew C Ahn, Muneesh Tewari, Chi-Sang Poon, Russell S Phillips

## Abstract

In the second of a two part series, Ahn and colleagues provide a practical discussion of how a systems approach will affect clinical medicine, using diabetes as an example.

*This is the second in a series of two articles that look at the lessons for clinical medicine from systems biology.*

In the first article in this series, we examined the reductionist approach that pervades medicine and explained how a systems approach (as advocated by systems biology) may complement it [ 1]. In order for a systems perspective to have any practical clinical significance, we must understand when a systems perspective is or is not helpful, and conversely when a reductionist approach is helpful. In addition, we must be able to envision how a systems perspective can be implemented to appreciate the potential benefits derived from its application. In this article, we address these issues and present a practical discussion of systems application to medicine.

## Indications for Systems Approach and Reductionism

Reductionism, as a guiding principle, is tremendously helpful and useful. The problem with reductionism stems not from its use but from the wrongful assumption that it is the only solution. Reductionism becomes less effective when the act of dividing a problem into its parts leads to loss of important information about the whole. For instance, a complex machine such as an airplane or a computer may be divided into smaller and smaller fragments, but at some point, the individual parts fail to impart consequential information about the machine's overall function. The primary side effect of a reductionist approach is that the act of reduction (from larger to smaller) disregards component–component interactions and the dynamics that result from them. Therefore, as a general rule, reductionism is less helpful for systems where interactions between components dominate the components themselves in shaping the system-wide behavior ( Table 1).

**Table 1:**
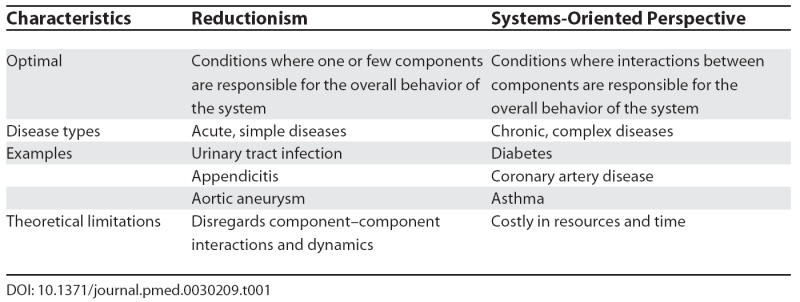
Application of Reductionism versus Systems-Oriented Perspective to Medicine

In clinical medicine, complex, chronic diseases such as diabetes, coronary artery disease, or asthma are examples where this rule may apply. In these examples, a single factor is rarely implicated as solely responsible for disease development or presentation. Rather, multiple factors are often identified, and the disease evolves through complex interactions between them. Consequently, a perspective in which the interactions and dynamics are centrally integrated into the analytical methods may be better suited. Systems perspectives, unlike reductionisms, focus on these interrelationships and therefore may be the optimal method for complex chronic diseases.

Where reductionism is helpful, when a systems approach is not, is when one or several components overwhelmingly influence the systems behavior. Diseases such as urinary tract infection, acute appendicitis, or aortic dissection are driven primarily by a single pathology amenable to a specific intervention. Arguably, these conditions would do poorly under a systems approach, where lengthy analysis and comprehensive data acquisition are often required. Reductionism works best when an isolatable problem exists and where a quick and effective solution is needed. For that reason, reductionism may generally be most effective for acute and simple diseases, whereas a systems approach may be most applicable to chronic and complex diseases.

## The Example of Diabetes

Given that a systems approach is likely applicable to complex chronic diseases, how might it influence the treatment of a complex disease such as diabetes? Research has shown that diabetes is a multidimensional disorder. Factors such as genetics, inflammation [ 2–7], PPAR-gamma [ 8], leptin [ 9], cortisol [ 10], diet [ 11], and body mass index, among others, have been implicated in some form with its pathogenesis. The fundamental distinctiveness of systems medicine is not just the recognition that these complex factors are important in disease management, but that they need to be incorporated in some meaningful way to treatment selection and delivery. The primary challenge tackled by systems scientists is the rigorous elucidation of how these multiple variables dynamically interact and how one can apply this understanding to affect the system and achieve a desirable end.

While this approach seems extremely complicated and difficult, the advent of computers and mathematical tools has opened avenues not deemed possible before. For the medical community, the more imminent hurdle may be our inability to envision and thus appreciate the potential benefits derived from the application of a systems approach. To obtain a glimpse of how systems principles will affect medicine, we consider three concepts central to systems medicine that are often overlooked through reductionist approaches: time, space, and context.

### Time.

Our present diagnosis of diabetes requires two separate documentations of fasting glucose over 6.9 mmol/L or a two-hour oral glucose tolerance test result above 11.1 mmol/L. The criterion relies on a measurement obtained at a single point in time, ideally eight hours after a meal or two hours after a glucose load. The theoretical disadvantage of this definition is that the diagnosis is established much after the underlying abnormality has begun. To use the analogy of a blocked sink—the problem is defined only when the water overflows, despite the fact that the draining problem has occurred some time beforehand. A more sensitive method for detecting a problem may be to evaluate the rate of change in the water level—whether the water level steadily increases with time or whether it fails to decrease in response to a large water input—in other words, to assess the dynamics of the variable of interest.

While this example is a gross oversimplification, it highlights a fundamental tenet of systems medicine, namely, that the dynamics may contain more revealing information about a system than static data alone. To apply this tenet to diabetes, one might assess the likelihood that glucose variability or the change in insulin levels over time may provide useful diagnostic information not otherwise obtained through traditional methods. Some evidence already exists to support this supposition. Healthy individuals show pulsatile insulin secretions of about six- to ten-minute periodic oscillations [ 12], whereas people with type 2 diabetes have impaired insulin oscillations [ 13], which also fail to entrain with repeated glucose infusions [ 14, 15]. Interestingly, impaired pulsatile secretions have been detected in metabolically normal yet predisposed individuals (first-degree relatives of people with type 2 diabetes) [ 16, 17], suggesting that these dynamic evaluations may be more sensitive in detecting beta-cell dysfunction than traditional methods [ 18]. Because of the promise of dynamic analysis in diabetes, many other methods have also been evaluated [ 19–21].

Because glucose levels are continually regulated through a dynamic balance between glucose-lowering factors (such as insulin) and glucose-elevating factors (such as glucagons, growth hormone, or epinephrine), the manner in which glucose varies over time may reflect the functional health of the relevant metabolic pathways. The premise is that glucose regulatory pathways are inextricably interconnected and that any dysfunction in the pathway is reflected in the glucose/insulin dynamics. The temporal changes of a variable contain hidden, useful information about the overall system. As a consequence, a systems approach to medicine will likely incorporate temporal variability into diagnosis and treatment in a way that reductionist medicine has never done before.

### Space.

When chemstick glucose levels are obtained, there is an implicit assumption that the geographic distribution of glucose is uniform: A chemstick in the right finger is equivalent to a chemstick from the left finger or a venous puncture reading from an antecubital region. But glucose, even plasma glucose, possesses spatial differences [ 22] that are frequently overlooked in clinical practice. The same can be said about insulin injections. Injections in the thigh are often considered just as effective as injections in the abdominal wall, despite evidence indicating that insulin absorption and distribution differ at different sites [ 23–25]. The problem confronted by clinical medicine is not so much the recognition that these variations occur, but rather the inability to incorporate spatial information into treatment or diagnostic decisions.

In systems theory, spatial variability, much like temporal variability, is valued for its potential to impart system-level information. Analytic tools such as diffusion equations and fluid dynamics are frequently used to evaluate the spatiotemporal patterns of various systems. Consequently, for diabetes, the application of systems principles may promote investigation and enhance understanding of the spatial variations of glucose and insulin within the human body. With proper tools and analytical techniques, we may someday be able to determine where insulin injections are most effective, how bodily glucose distribution can predict risk of diabetes, and how certain foods lead to unhealthy overstimulation of certain susceptible beta-islet cells. The one caveat, however, is that spatial information, such as glucose distribution, is difficult to acquire and may explain why spatial variability of glucose remains largely unexplored. Nevertheless, a systems approach may provide a much-needed conceptual tool for the study of spatial influence in medicine and thus may inform health providers where optimal solutions exist.

### Context.

One of the principal challenges for medical practitioners will be to curb our instinctive inclination to focus on disease rather than the individual. In diabetes, for instance, we are inclined to focus on the symptom—hyperglycemia—and to deliver treatments aimed directly at lowering glucose. While this approach is highly effective, a systems approach to medicine may redirect our attention away from the elevated glucose per se, toward the contextual milieu that fosters it. Dietary habits, sleeping behavior, immune system, genetics, psychiatric condition, medical comorbidities, and other factors can be systematically integrated into a physician's selection and delivery of treatment. The individual, not the disease, achieves central importance in systems medicine.

However, with a systems perspective, will treatments truly change? How can a patient with diabetes *not* receive glucose-lowering agents? How will “disease” be conceptualized, and will it be defined any differently? Fortunately, studies in systems biology have addressed similar questions and provide two important lessons for clinical medicine: (1) complex diseases may represent many different processes and (2) complex diseases may have varied and sometimes unintuitive treatments.

Systems biology's first lesson for clinical medicine can be derived from the RNA expression profiles of diffuse B cell lymphoma. The lymphoma's genetic profile yielded an unexpected, yet important discovery—namely that for a disorder once considered a single entity, at least three different genetic profiles exist: germinal-center B cell–like, activated B cell–like, and type 3 diffuse large B cell lymphoma [ 26]. Genetic profiles of other disorders—such as breast cancer [ 27], non-small-cell lung carcinoma [ 28], and acute lymphoblastic leukemia [ 29]—have similarly shown the existence of multiple subtypes. The conceptual breakthrough afforded by these findings is the idea that seemingly single phenotypic entities can have multiple etiologic or pathologic processes. For clinical medicine, this may mean that diseases such as diabetes actually represent many different processes that do not become apparent until the composite factors (i.e., the context) are considered. Therefore, two patients with type 1 diabetes who have identical presentations may nevertheless have different pathogenic processes, and thus should be regarded differently.

The study of diffuse B cell lymphoma also showed that the three identified subtypes have varied prognoses and responsiveness to chemotherapy [ 26, 30, 31]. Consequently, systems biology's lesson can be extended one step further, to suggest that not only do different processes exist for a specific disease but that each process should be treated or handled differently. This notion encourages the personalization or individualization of medicine. One patient with type 2 diabetes may respond best to insulin, for example, while another may not. As a corollary to this statement, some patients with diabetes may not require glucose-lowering agents at all, but instead may benefit from a less obvious treatment. The determination of these optimal treatments will rest on the rigorous evaluation of the complex factors inherent in each and every patient.

## Systems Medicine in Practice

Systems medicine, as we see, begins to explore medicine beyond linear relationships and single parameters. Systems medicine involves multiple parameters obtained across multiple time points and spatial conditions to achieve a holistic perspective of an individual. The clinical practice that results from this paradigm will be distinctly different from the status quo, particularly for complex diseases, as shown by our example of diabetes ( Table 2). In general, treatments within systems medicine can be characterized by several distinguishing features ( Figure 1).

**Table 2:**
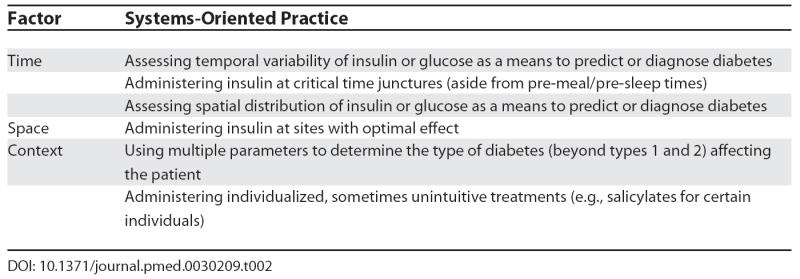
Approaching Diabetes within a Systems Perspective

**Figure 1:**
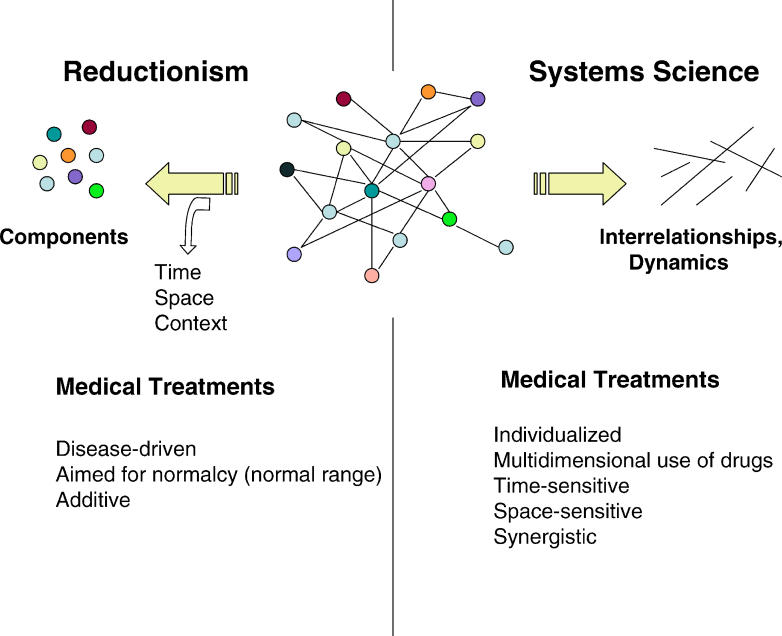
Medical Treatments: Reductionism versus Systems Science Treatment differences stem from divergent problem-solving tactics. Reductionism focuses on components and, in the process, can lose information about time, space, and context. Systems science focuses on the interactions and dynamics and spends less time studying the individual components.

### Individualized treatments.

Instead of formulating treatments according to disease, a systems clinician may prescribe treatments specifically targeted to individuals and their present conditions.

### Minimized interventions.

Treatments can deliver the “biggest bang for the buck” so that the least invasive intervention may yield the greatest system-wide benefit, maximize the body's self-healing abilities, and minimize side effects.

### Multidimensional uses of medications.

Medications may be used for unintended purposes because nonlinear, unintuitive relationships exist between pathogenic factors and disease. In diabetes, for example, evidence suggests the benefits of salicylates for glucose control in certain individuals [ 32–34].

### Time-sensitive treatments.

The human body, like most living systems, has cyclical variations that may affect treatment efficacy. To maximize effectiveness, treatments can be delivered at selective times. Cancer chronotherapy is a working example: chemotherapeutic agents given on a timed regimen are more effective than a standard treatment approach [ 35–37].

### Space-sensitive treatments.

The efficacy of certain treatments may depend on where the treatment is delivered. Future treatments may be localized to a specific part of the body to maximize system-wide efficacy.

### Synergistic treatments.

Use of more than one treatment or modality can be given so that the effects are synergistic and not antagonistic or merely additive.

### Probabilistic forecasting.

The probability of the success or failure of a particular treatment may be calculated specifically for an individual.

### Temporary treatments.

Chronic treatments may be unnecessary. In systems biology, biological systems are understood to assume certain dynamic states—or “attractor states” [ 38, 39]. Disease may represent certain attractor states, while health may represent others. If so, it is theoretically possible to affect the system dynamics and transform a diseased state to a healthy one through limited interventions [ 40]. Because these states are effectively stable, chronic treatments may be unnecessary.

These practices and concepts are not new to medical systems. Medical traditions such as traditional Chinese [ 41], Native American [ 42], and certain Western medicines have for centuries incorporated these practices in their care of patients, mainly due to the philosophical belief that the world (including humans) is dynamic and interactive. Unlike modern systems medicine, however, human intuition and observation rather than mathematical/computational tools served as the basis for advancing medical knowledge.

## Barriers to Systems Medicine

Widespread benefits of systems medicine will not be realized until six key barriers are overcome. First, the network relationships will need to be elaborated in detail. In diabetes, for instance, we lack the in-depth knowledge of how diet, inflammation, PPAR-gamma, genetics, and other factors interrelate and influence each other's behavior. Secondly, a feasible and cost-effective means to acquire comprehensive data will need to be developed. Clinical medicine at the present moment lacks an equal to the DNA array chip that enables numerous parameters to be economically obtained at once. In addition, we lack the means to obtain measures across multiple temporal and spatial conditions without causing patient inconvenience and excessive costs. Third, the optimal balance between too little information and too much information needs to be established. Often, accumulation of information beyond a certain point may contribute to costly expenditures without adding any effective understanding of the system. Fourth, the analytical tools for determining how to affect biological networks and obtain the desired effect need to be perfected. How should we calculate the needed adjustments to our patients' diets to minimize their pancreatic beta-cell loss? The mathematical and computational tools are available but still imperfect. Fifth, the theoretical and experimental methods should be effectively integrated in order for systems science to truly advance. Finally, complex analysis is inherently a long-term, broad-based investment. To investors and researchers accustomed to immediate, predictable results, this consideration may present the greatest barrier, causing many to doubt whether the not-so-apparent benefits merit further financial or temporal commitment.

Despite these challenges, the realization of systems medicine may not be as distant as many may think. Already a computer program called Archimedes has been developed for the complex modeling of diabetes and predicts diabetes-related clinical outcomes with uncanny accuracy [ 43, 44]. Archimedes is just a sample of the many more systems-level programs that will likely emerge within the next five to ten years.

## Conclusion

Systems medicine encompasses a broad scope of topics, many of which have been untouched in this two part series. Examples include scaling, stochasticity, attractor states, plasticity, systems definition of health, and many others. The challenges of incorporating systems science into medicine are difficult but not insurmountable. In fact, systems biologists, who deal with thousands of genes and proteins, may arguably be confronted with a much more daunting task. Nevertheless, systems biologists have recognized the necessity of a systems perspective. It is time that physicians, clinical researchers, physiologists, and epidemiologists did the same. The specific task to be faced is the system-level understanding of human health and disease at the organ, organism, and community level. This effort has great potential for the advancement of medicine.
